# Selection and validation of appropriate reference genes for RT–qPCR analysis of *Nitraria sibirica* under various abiotic stresses

**DOI:** 10.1186/s12870-022-03988-w

**Published:** 2022-12-17

**Authors:** Aishuang Hu, Xiuyan Yang, Jianfeng Zhu, Xiuping Wang, Jiaxin Liu, Jiping Wang, Haiwen Wu, Huilong Zhang, Huaxin Zhang

**Affiliations:** 1 Institute of Ecological Protection and Restoration, Chinese Academy of Forestry, 10091 Beijing, China; 2 The Comprehensive Experimental Center of Chinese Academy of Forestry in Yellow River Delta, 257000 Dongying, China; 3 Institute of Coastal Agriculture, Hebei Academy of Agriculture and Forestry Sciences, 063299 Tangshan, China; 4 Hebei saline-alkali Land Greening Technology Innovation Center, 063299 Tangshan, China

**Keywords:** *Nitraria sibirica* Pall., geNorm, NormFinder, BestKeeper, Comparative ΔCt, RefFinder, Salt stress

## Abstract

**Background:**

*Nitraria sibirica* Pall. is a halophytic shrub with strong environmental adaptability that can survive in extremely saline-alkali and drought-impacted environments. Gene expression analysis aids in the exploration of the molecular mechanisms of plant responses to abiotic stresses. RT–qPCR is the most common technique for studying gene expression. Stable reference genes are a prerequisite for obtaining accurate target gene expression results in RT–qPCR analysis.

**Results:**

In this study, a total of 10 candidate reference genes were selected from the transcriptome of *N. sibirica*, and their expression stability in leaves and roots under different treatment conditions (salt, alkali, drought, cold, heat and ABA) was evaluated with the geNorm, NormFinder, BestKeeper, comparative ΔCt and RefFinder programs. The results showed that the expression stability of the candidate reference genes was dependent on the tissue and experimental conditions tested. *ACT7* combined with *R3H*, *GAPDH*, *TUB* or *His* were the most stable reference genes in the salt- or alkali-treated leaves, salt-treated roots and drought-treated roots, respectively; *R3H* and *GAPDH* were the most suitable combination for drought-treated leaves, heat-treated root samples and ABA-treated leaves; *DIM1* and *His* maintained stable expression in roots under alkali stress; and *TUB* combined with *R3H* was stable in ABA-treated roots. *TBCB* and *GAPDH* exhibited stable expression in heat-treated leaves; *TBCB*, *R3H*, and *ERF3A* were stable in cold-treated leaves; and the three most stable reference genes for cold-treated roots were *TBCB*, *ACT11* and *DIM1*. The reliability of the selected reference genes was further confirmed by evaluating the expression patterns of the *NsP5CS* gene under the six treatment conditions.

**Conclusion:**

This study provides a theoretical reference for *N. sibirica* gene expression standardization and quantification under various abiotic stress conditions and will help to reveal the molecular mechanisms that confer stress tolerance to *N. sibirica*.

**Supplementary Information:**

The online version contains supplementary material available at 10.1186/s12870-022-03988-w.

## Background

*Nitraria sibirica* Pall. is a typical salt-diluting halophyte shrub that can adapt to diverse adverse stresses, such as high salinity, alkali, drought and extreme temperatures. Therefore, *N. sibirica* is often used as a pioneer tree for windbreaks, sand fixation and the improvement of heavily saline-alkali soils [1, 2]. As a sessile plant that grows in extreme environments, *N. sibirica* has had to develop a series of complex mechanisms to resist or adapt to adverse environmental conditions. In addition, *N. sibirica* can also serve as an economic plant in saline–alkali areas due to its medicinal value and edible fruit [3]. In recent years, the physiological mechanisms of resistance to stress in *N. sibirica* have been studied, including the distribution of Na^+^ and K^+^, osmotic adjustment and changes in antioxidase activity [4–6]. However, knowledge of the molecular mechanism of *N. sibirica* stress resistance is relatively insufficient.

Gene expression analysis is the foundation for revealing gene function and can help to reveal the molecular mechanisms involved in plant stress resistance. RT–qPCR (real-time quantitative polymerase chain reaction) is the most common technique used to analyse gene expression because it is highly sensitive, cost-effective, reproducible and specific. RT–qPCR has been widely used to investigate the expression patterns of genes in diverse organisms or under different environmental conditions [7–9]. However, the accuracy and reliability of RT–qPCR results are affected by many factors, such as the quality of the RNA, the efficiency of cDNA synthesis, and the efficiency of amplification [10, 11]. To obtain accurate gene expression results, the data generated by RT–qPCR should be normalized with the use of appropriate reference genes [7, 11]. Housekeeping genes that maintain the cytoskeleton or participate in basic biochemical metabolism, such as *actin* (*ACT*), *β-tubulin* (*TUB*), *elongation factor 1-α* (*EF-1α*) and *glyceraldehyde 3-phosphate dehydrogenase* (*GAPDH*), are usually selected as internal reference genes [12–14]. The expression levels of reference genes should ideally be consistent in different tissues and under different environmental conditions [15, 16]. However, numerous studies have found that the stability of reference gene expression is different in different species, tissues and experimental treatments [17, 18]. For example, *ACT* was a stable reference gene in *Platycladus orientalis* [19] but unstable in teak (*Tectona grandis* L.f.) [20]. *Ubiquitin conjugating enzyme* (*UBC*) and *GAPDH* were the most stable reference genes in the flowers of *Iris germanica* L. ‘00246’ and ‘Elizabeth’, while the most stable reference genes in the flowers of *Iris germanica* L ‘2010200’ were *TUB* and *UBC* [21]. In *Glehnia littoralis*, *expressed protein 1* (*EXP1*) and *serine/threonine-protein phosphatase PP2A* (*PP2A*) were the most stable reference genes under salt stress, while *cyclophilin 2* (*CYP2*) and *α-TUB* were the most stable genes under MeJA treatment [22]. In addition, it has been reported that some newly characterized genes may be more stable than traditional housekeeping genes. Under salt and drought stress, the expression of the new reference genes *RG1*, *RG3* and *RG5* in poplar was more stable than that of *18S ribosomal RNA* (*18S rRNA*), *Actin* and *ubiquitin* (*UBQ*) [23]. The expression stability of unigene 14,912 was higher than that of *18S rRNA*, *GAPDH* and *His3* in annual ryegrass (*Lolium multiflorum*) under saline-alkali stress [24]. These studies show that there is not a universal reference gene for all experiments and all plant species [16, 25]. It is necessary to systematically screen and identify internal reference genes before carrying out RT–qPCR experiments in different species, treatment conditions and different tissues. To date, there have been no reports on the screening and evaluation of appropriate reference genes in *N. sibirica* under abiotic stress.

The objective of the present study was to determine the optimal reference genes for RT–qPCR analysis in *N. sibirica* under various treatment conditions, such as salt, alkali, drought, heat and cold stresses and ABA treatments. To accomplish this objective, we screened *N. sibirica* transcriptome data for candidate reference genes, and five statistical algorithms (geNorm [26], NormFinder [10], BestKeeper [27], comparative ΔCt [28] and RefFinder [29]) were applied to evaluate their expression stability for normalization. In addition, the expression of *Δ*^1^*-pyrroline-5-carboxylate synthetase* (*NsP5CS*) was analysed to demonstrate the effectiveness of the selected reference genes. Our work will facilitate further studies of gene expression in *N. sibirica* and will accelerate the understanding of the molecular mechanisms underlying stress-induced responses in *N. sibirica*.

## Results

### Primer specificity and amplification efficiency test of candidate reference genes

A total of 10 genes were selected from transcriptome data and used as candidate reference genes. qPCR primers were designed based on these sequences. All primers showed a signal and clear band of the expected size, and there was no primer-dimer formation observed on gel electrophoresis (Fig. S1), indicating the specificity of the primer pairs. The single peaks presented in the melting curve assays of each gene further verified the specificity of the primer pairs (Fig. S2). The amplification efficiency of the 10 candidate reference genes ranged from 89.67% (*His*) to 113.81% (*ACT11*), and the correlation coefficients (R^2^) varied from 0.984 to 0.999 (Table 1). These results indicated that the primers of the 10 genes met the standards for qPCR and could be used in subsequent experiments. The details of the candidate reference genes, primer sequences, amplification lengths and efficiencies, and correlation coefficients are shown in Table 1.

**Table 1:** Ten candidate reference genes and primer sequences (forward and reverse 5’-3’) used in the study

Gene symbol	Gene description	GenBank accession	Forward/Reverse primer(5’-3’)	Primers Tm (℃)	Amplicon length (bp)	RT–qPCR Efficiency	Correlation Coefficient
*ACT7*	*actin-7*	actin-7	GGAATCCACGAGACCACCTACA	61.2	219	100.71	0.998
			GATTGATCCTCCGATCCAGACA	61.1			
*ACT11*	*actin-11*	ON855356	CAGGCTGTTCTTTCGCTTTAC	57.4	228	113.81	0.998
			AATGTCCCTCACAATTTCACG	57.4			
*TUB*	*β-tubulin*	ON855357	CAGAGAGGAGTATCCAGACCGTAT	58.7	218	98.35	0.999
			GGTGGTTAAGATCGCCAAATGTAG	62.2			
*DIM1*	*dimethyladenosine transferase*	ON855358	AAGAACAATTTCCGTCCTCC	55.8	292	103.45	0.984
			CACTACCACATTCCACCTCC	54.7			
*TBCB*	*tubulin-folding cofactor B*	ON855359	TGGTATCTCAGAACCCATCAGC	59.1	246	92.14	0.997
			GTGGAGGACAGTCAAAGTAACG	56.6			
*ERF3A*	*eukaryotic peptide chain release factor GTP-binding subunit ERF3A*	ON855360	CGCCACCACAAGAAACCAAA	61.7	297	98.96	0.998
			TCTCATCCTCCGAGTCAACA	55.7			
*GAPDH*	*glyceraldehyde-3-phosphate dehydrogenase*	ON855361	CTGTTCACTCCATCACTGCCAC	60.2	282	103.14	0.998
			TCATTTTTCCCTCAGATGCCTC	60.8			
*EF-1α*	*elongation factor 1-α*	ON855362	CCTGGACATCGTGACTTTATC	54.5	277	106.38	0.996
			GGTTGTATCCGACCTTCTTTAG	55.5			
*R3H*	*R3H domain-containing protein*	ON855363	TCGTCTAACCATTCTGCGGA	59	178	102.78	0.995
			CCCAAGCCCTTCTAAACCAC	58.8			
*His*	*Histone*	ON855364	CCTCACCGTTACCGCCCT	59.46	182	89.67	0.999
			GCCTCAGCTGCCTCTTGC	59.46			
*P5CS*	*Δ1-Pyrroline-5-carboxylate synthetase*	ON855365	TGCCCTTTGGGTGTCCTCTT	61.7	132	94.32	1.000
			TGCATTTGATCGCTTAGCTTCT	59.8			

### Expression levels of candidate reference genes in leaves and roots

**Fig. 1:**
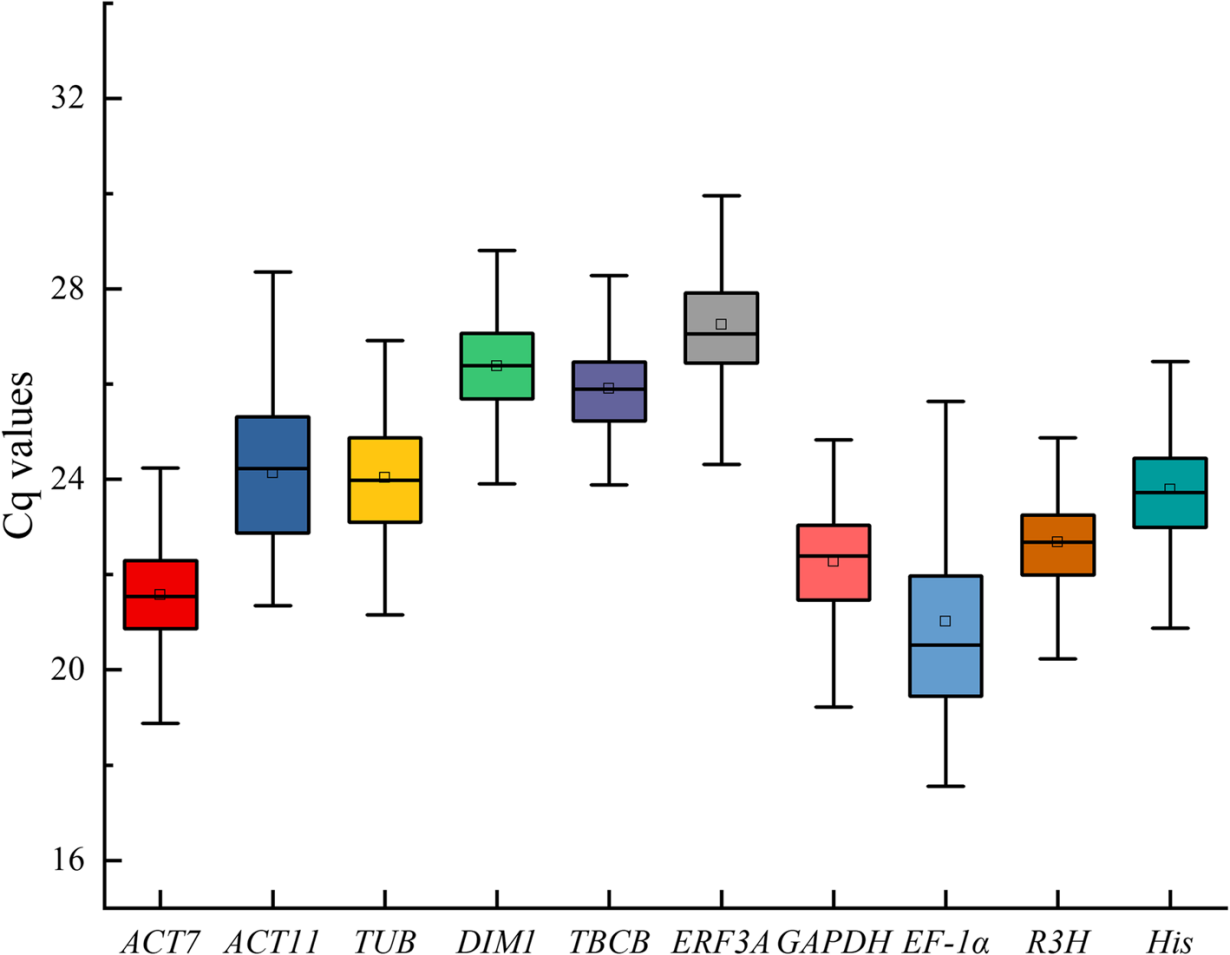
The raw Cq values for 10 candidate reference genes in all samples of *N. sibirica*. The box indicates the 25th to 75th percentiles, the line across the box represents the median, the inner square represents the mean, and the whiskers represent the maximum and minimum values

The quantification cycle (Cq) value reflects the mRNA transcript level. Reference genes with higher Cq values are considered to have lower expression abundance. Based on RT–qPCR, we determined the expression levels of 10 candidate reference genes in root and leaf tissues under salt, alkali, drought, cold, heat and ABA treatments. As shown in Fig. 1, the Cq values of the 10 candidate reference genes varied from 17.56 (*EF-1α*) to 29.95 (*ERF3A*), indicating a wide range of expression abundance. *EF-1α* and *ERF3A* were the most [mean Cq of 21.02 ± 2.46 (standard deviation, SD)] and least (mean Cq of 27.25 ± 1.66) expressed genes. In addition, it was observed that *TBCB*, *R3H* and *DIM1* had relatively small SD values of 0.87, 0.99 and 1.01, respectively, whereas *EF-1α* and *ERF3A* showed relatively high variation, with SD values of 2.46 and 1.66, respectively (Fig. 1, Table S1). Further analysis of the distribution of Cq values showed that the Cq values of the 10 candidate reference genes varied differently in different tissues and treatments. For example, *ACT11* and *TUB* had Cq values of 23.06 ± 0.93 and 23.48 ± 1.06 in leaves, respectively, but their Cq values in roots were 25.22 ± 0.90 and 24.72 ± 1.61, respectively (Fig. S3). The Cq value of *EF-1α* was 19.66 ± 1.13 under salt treatment, but the Cq value under heat treatment was 22.80 ± 3.14 (Fig. S4). Therefore, screening suitable reference genes for specific experimental treatments and tissues is needed.

### Expression stability of candidate reference genes

Four specialized analytical tools, geNorm, NormFinder, BestKeeper and the comparative ΔCt method, were used to examine the expression stability of the 10 candidate reference genes. Subsequently, the RefFinder tool was employed to evaluate the expression stability of all these candidate reference genes and select the most suitable genes.

#### geNorm analysis

**Fig. 2:**
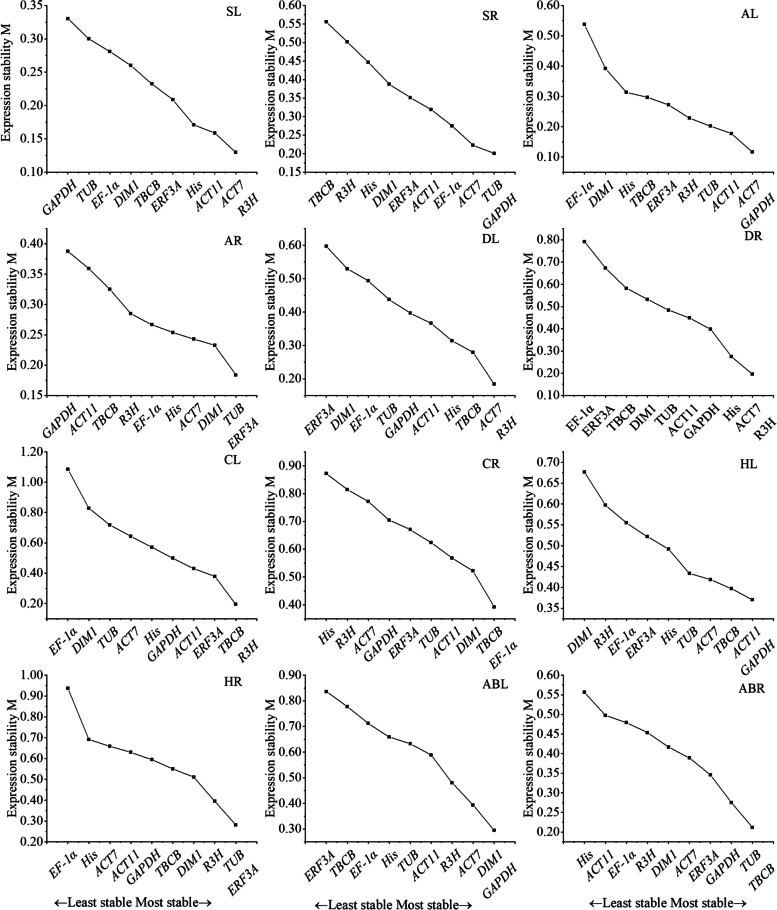
Expression stability values (M) of 10 candidate reference genes validated via the geNorm program. The least stable genes with higher M-values are on the left, and the most stable genes with lower M-values are on the right. SL and SR: Salt-treated leaves and roots, respectively; AL and AR: alkali-treated leaves and roots, respectively; DL and DR: drought-treated leaves and roots, respectively; CL and CR: cold-treated leaves and roots, respectively; HL and HR: heat-treated leaves and roots, respectively; ABL and ABR: abscisic acid-treated leaves and roots, respectively

**Fig. 3:**
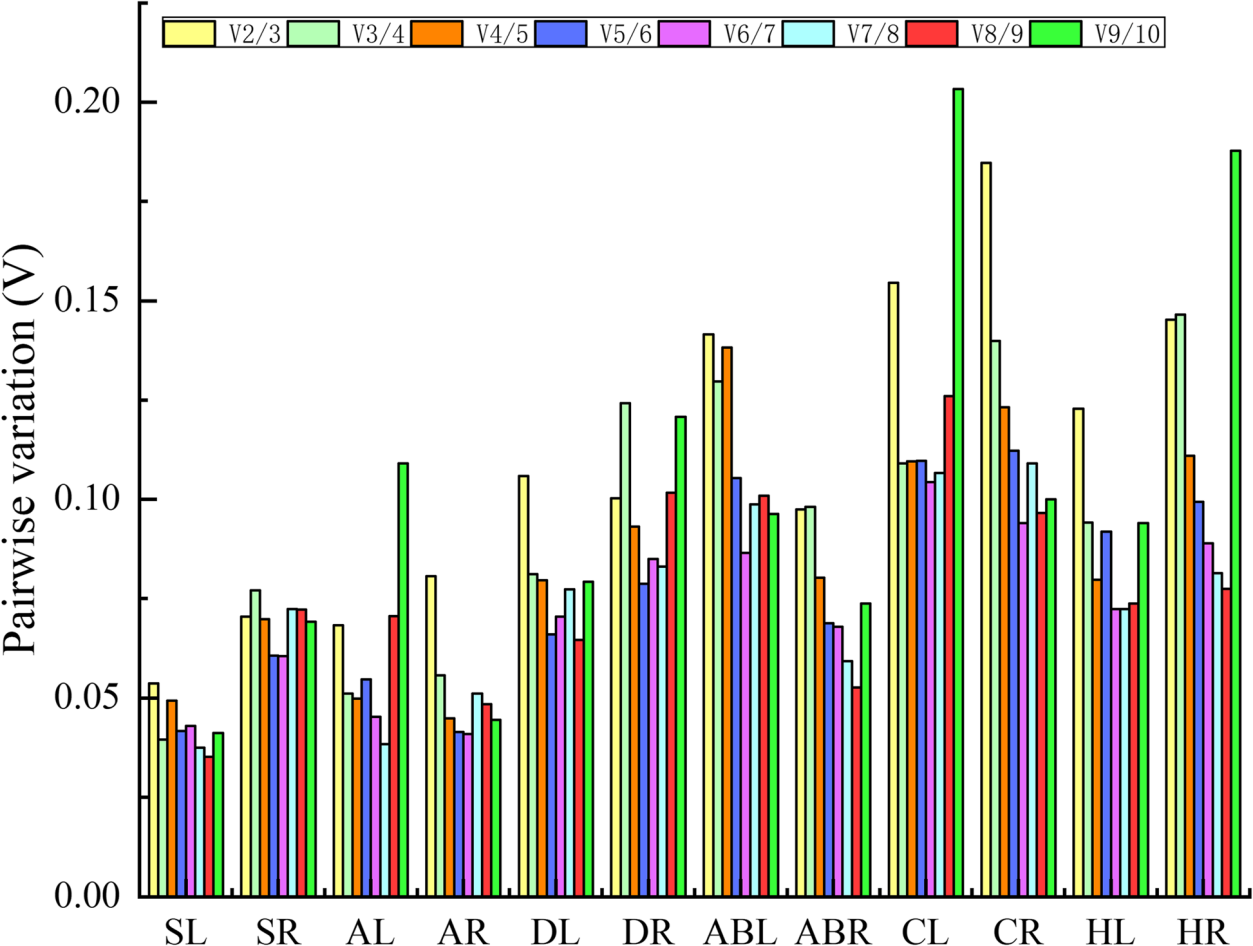
Pairwise variation (V) of the 10 candidate reference genes calculated by geNorm to determine the optimal number of reference genes for accurate normalization. A cut-off of 0.15 (Vn/n + 1 value) is usually applied

geNorm evaluated the stability of all 10 candidate reference genes using the M value (reference expression stability measure). The default threshold of M is 1.5. A lower M value indicates more stable gene expression [26]. As illustrated in Fig. 2, the M values of all 10 candidate reference genes in every treatment were lower than the default limit of 1.5, and all the candidate reference genes had different levels of stability under different treatments. *ACT7* and *R3H*, which had the same M values, were the most stable genes for salt-treated leaves (SL), drought-treated leaves (DL) and drought-treated roots (DR); *TUB* showed good stability for salt-treated roots (SR), alkali-treated roots (AR), heat-treated roots (HR) and abscisic acid-treated roots (ABR); and *GAPDH* was one of the most stable genes in SR, alkali-treated leaves (AL), heat-treated leaves (HL) and abscisic acid-treated leaves (ABL). *EF-1α* and *His* were determined to be the least stable reference genes across treatments. geNorm software also provides information on the optimum number of reference genes to be used in an experiment based on the pairwise variation between ranked genes (Vn/n + 1), and a cut-off of 0.15 (V value) is usually applied to determine whether an additional reference gene needs to be added [26]. If Vn/n + 1 > 0.15, then n + 1 reference genes need to be used; otherwise, only n reference genes are needed. As shown in Fig. 3, the V2/3 values for the SL (0.054), SR (0.071), AL (0.068), AR (0.081), DL (0.106), DR (0.100), ABL (0.142), ABR (0.097), HL (0.123) and HR (0.145) samples were lower than 0.15 (Fig. 3), indicating that two reference genes would be sufficient for the accurate normalization of these treatment samples. The V2/3 values for cold-treated leaves (CL) and cold-treated roots (CR) exceeded 0.15, while the V3/4 values were 0.110 and 0.140, respectively, indicating that three reference genes were needed.

#### NormFinder analysis

**Fig. 4:**
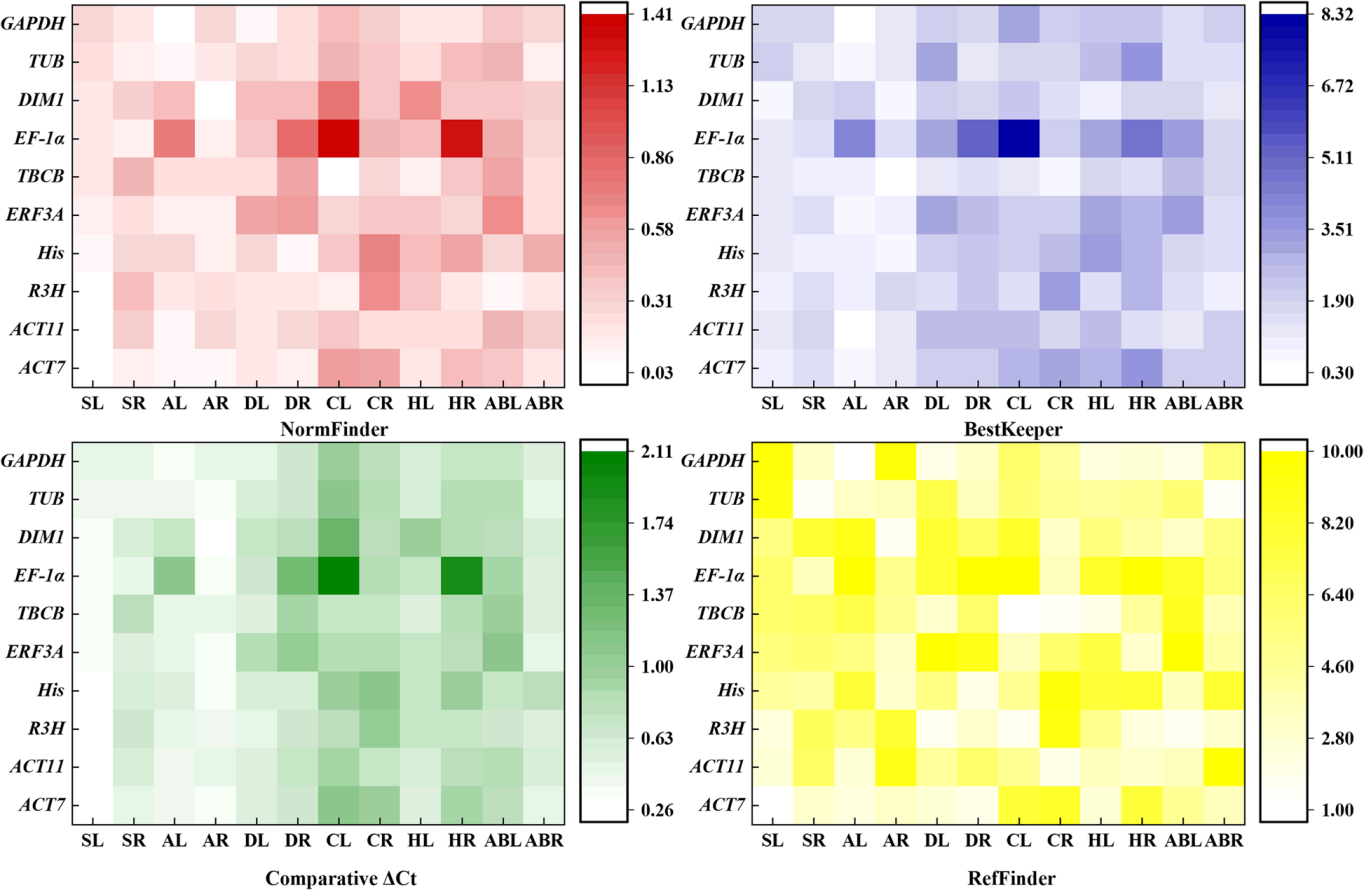
Stability analysis of the 10 candidate reference genes by NormFinder, BestKeeper, comparative ΔCt and RefFinder. Within a pane, the smaller the value, the lighter the colour, and the higher the stability of the candidate reference genes

NormFinder software directly evaluates the stability of reference genes according to the variance within and between groups, with lower values indicating higher stability [10]. The stability values of the candidate reference genes in each treatment are shown in Fig. 4 and Table S2. The stability rankings of the 10 reference genes under different experimental conditions were not completely consistent. For example, *ACT7* was the most stable reference gene in the SL samples, while *TUB* was ranked first in the SR and ABR samples, and *GAPDH* was the top ranked gene for the AL, DL and HR samples. The stability ranking of the 10 candidate reference genes generated with NormFinder was slightly different from that produced by geNorm in most of the samples. For example, *His* and *ACT11* were the top ranked reference genes for the DR and CR samples in the NormFinder analysis, while they ranked third and fourth, respectively, in the geNorm analysis.

#### BestKeeper analysis

BestKeeper evaluates the stability of gene expression by calculating the standard deviation (SD) and coefficient of variation (CV) of the Cq values. A smaller SD and CV indicate better stability of gene expression. If SD > 1, the gene was considered unstable [27]. The analysis results are listed in Fig. 4 and Table S3. *GAPDH* was the most stable gene in the AL samples. *His* and *TUB* were ranked as the most stably expressed genes in the SR and DR samples, respectively, but exhibited the lowest stability in the HL and SL samples, respectively. *DIM1* was the most stable reference gene in the SL and HL samples, and *ACT11* was the most stable reference gene in the HR and ABR samples. For the AR, DL, CL and CR samples, the most stable gene was *TBCB*. *EF-1α* was ranked as the least stably expressed gene in most of the samples, including the AL, AR, DL, DR, CL, HL, HR and ABL samples. Overall, the stability ranking of the 10 candidate reference genes generated with BestKeeper was different from that of geNorm and NormFinder.

#### Comparative ΔCt analysis

This method evaluated gene expression stability by calculating the mean standard deviation (SD) value of each gene. Here, the smaller the value is, the higher the stability [28]. In SL, AL, AR, DR, HL, and ABR samples, *ACT7* was one of the two best reference genes for gene normalization. For ABL, DL, CL, and HR samples, one of the two most stable reference genes was *R3H*. In addition, the comparative ΔCt analysis also showed that *EF-1α* was the least stably expressed gene in multiple samples, such as AL, DR, CL and HR samples (Fig. 4 and Table S4).

#### RefFinder analysis

To reduce the influence of the limitations of a single algorithm, comprehensive stability rankings of the candidate reference genes were determined with the RefFinder program (https://www.heartcure.com.au/reffinder/). The ranking of genes was computed as the geometric mean, and a lower geometric, a higher stability []. As shown in Fig. 4 and Table S5, *ACT7* combined with *R3H*, *TUB*, *GAPDH* or *His* were the two most stable reference genes in the SL, SR, AL, and DR samples; *R3H* and *GAPDH* were suggested to be the most suitable combination for DL, ABL, and HR samples; for AR samples, the two most stable reference genes were *DIM1* and *His*; and *TUB* and *R3H* were the most stable reference genes for ABR samples. *TBCB* and *GAPDH* were the most stable reference genes for HL samples; *TBCB*, *R3H*, and *ERF3A* were found to be the three most stable reference genes in CL samples; and the three most stable reference genes for CR samples were *TBCB*, *ACT11* and *DIM1*. *EF-1α* was the least stable reference gene in most samples.

#### Reference gene validation

**Fig. 5:**
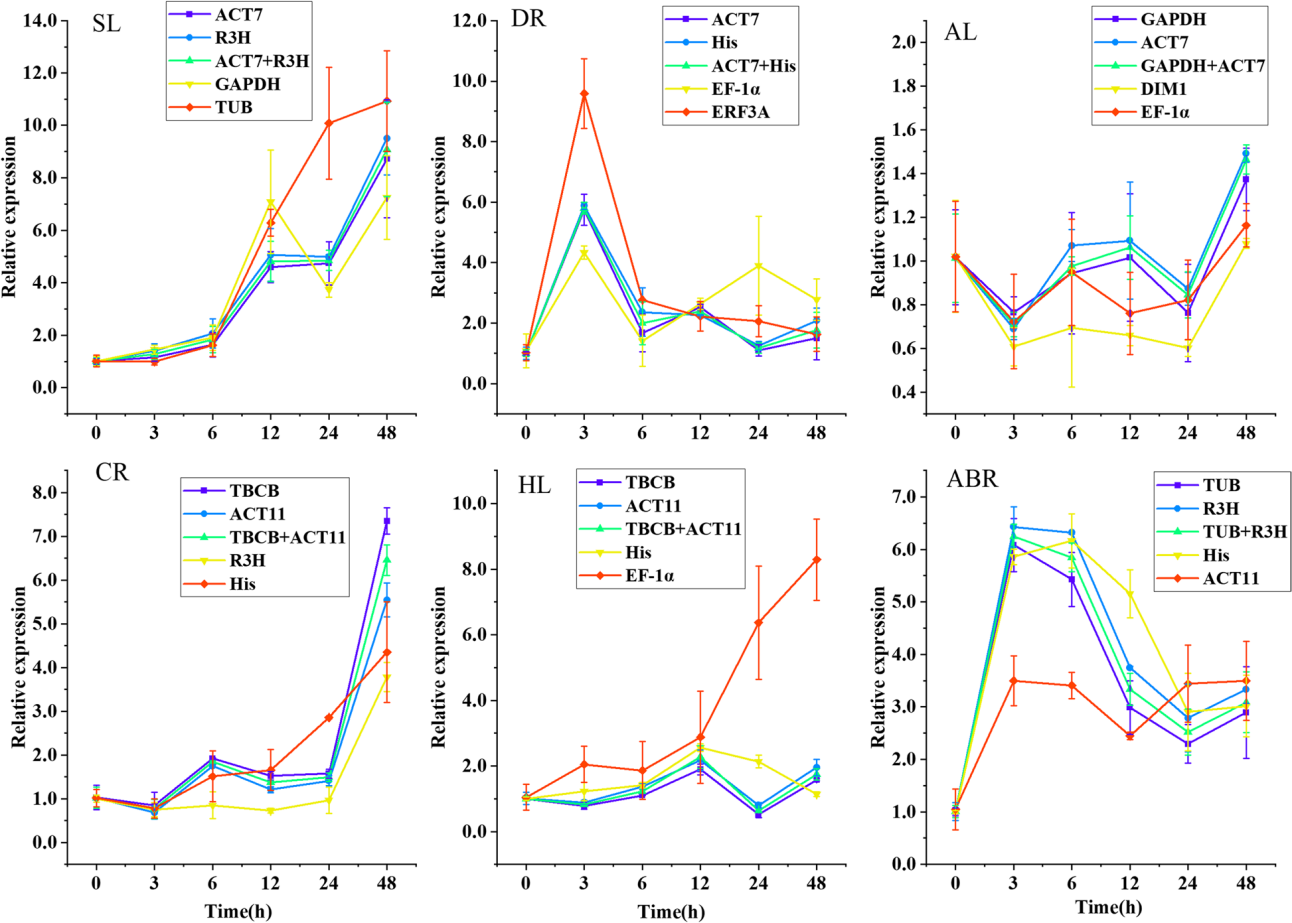
Relative expression of *NsP5CS* at 0, 3, 6, 12, 24, and 48 h following stress treatment using the selected reference genes for normalization. The validated reference gene(s) used as normalization factors were the two most stable reference genes (alone or in combination) and the two most unstable reference genes in different treatment groups. Error bars show the standard error calculated from three biological replicates

To verify the results generated through the analyses described above, the expression pattern of *NsP5CS* was examined in SL, DR, AL, CR, HL and ABR samples. P5CS is a rate-limiting enzyme in proline biosynthesis and plays an important role in controlling plant stress tolerance [30, 31]. The two most stable reference genes (alone and in combination) and the two most unstable reference genes based on the comprehensive ranking results for each sample set were used in the validation test. As shown in Fig. 5, the relative expression levels of *NsP5CS* normalized with the two most stable reference genes in combination showed significant changes in SL, DR, CR and ABR samples and different trends among different sample sets. In the SL and CR samples, *NsP5CS* was continuously induced, and its relative expression reached the highest level at 48 h, with values of 9.08 and 6.45, respectively. In DR samples, *NsP5CS* was rapidly induced and reached a maximum expression level at 3 h and then fluctuated at a lower level with time. *NsP5CS* was also rapidly induced and reached a maximum value at 3 h and remained at a higher value at 6 h for ABR samples. The expression patterns of *NsP5CS* normalized with the two most stable reference genes alone or in combination exhibited consistency in SL, DR, CR and ABR samples. However, the expression patterns of *NsP5CS* were significantly different when unstable reference genes were used. In the AL and HL samples, the expression level of *NsP5CS* varied less, but there were also similar expression patterns of *NsP5CS* normalized by the two most stable reference genes (alone or in combination) and were significantly different by the two unstable reference genes.

## Discussion

RT–qPCR is one of the primary methods used to detect gene expression and thereby help to reveal the response mechanism of plants under different stresses [32]. The accuracy of gene expression data obtained by RT–qPCR analysis relies on the use of reference genes [33]. The use of a stable reference gene(s) for normalization ensures that the target gene expression data generated by RT–qPCR are reliable. In contrast, the use of an inappropriate internal reference gene will lead to inaccurate results [34]. In this study, a total of 10 reference genes were selected from the transcriptome data of *N. sibirica* to assess their expression stability in leaves and roots under salt, alkali, drought, cold, heat and ABA treatment. As shown in Table 1 and S1, the 10 primer pairs were specific, generating a single peak in the dissolution curve. The amplification efficiency of the 10 candidate genes ranged from 89.67 to 113.81%, and the R^2^ was > 0.98, within the practical range [22, 35, 36]. Hence, the PCR conditions were acceptable. The mean Cq of the 10 candidate reference genes varied from 21.02 to 27.25 (Fig. 1). Considering that genes with a moderate expression level (a Cq value of 15 to 30, for instance) provide the most accurate normalization [37], the genes selected in this study met this requirement for use in normalization. Moreover, as a narrow distribution range tends to represent low variability, *TBCB*, *R3H* and *DIM1* should be considered stable reference genes, with SD values of 0.87, 0.99 and 1.01, respectively. The Cq value of the genes is for the total sample pool. Considering that the reference genes have different changes in different tissues and experimental conditions (Fig. S3, Table S1), the stability of genes needs to be analysed separately for specific tissues and specific treatments, and more tools are needed.

geNorm, NormFinder, BestKeeper and comparative ΔCt are algorithms that have been widely used for determining the stability of reference genes based on statistical analysis [16, 18, 24]. In the present study, the rankings generated by geNorm, NormFinder and comparative ΔCt were more similar to each other and different from those obtained by BestKeeper. For example, the results of geNorm, NormFinder and comparative ΔCt showed that *ACT7* and *TUB* were the most stable reference genes in SL and SR samples, while BestKeeper ranked third in stability. A similar difference between BestKeeper and other programs has also been reported in *Nitraria tangutorum* [13], *Poa pratensis* L. [38] and *Maiyuanjinqiu* [39], likely the result of the different algorithms that these programs employ [39, 40]. RefFinder is a comprehensive evaluation program that uses data from geNorm, NormFinder, BestKeeper and comparative ΔCt to calculate the geometric mean of candidate reference genes and generate a comprehensive stability ranking [41, 42]. In this study, five programs (geNorm, NormFinder, BestKeeper, comparative ΔCt and RefFinder) were used to evaluate the stability of candidate reference gene expression in *N. sibirica*. Similar methods have been used in previous studies for many species, such as *Eriobotrya japonica* [42], *Angelica decursiva* [16] and wheat (*Triticum aestivum* L.) [43]. It has been reported that two or more stable reference genes are necessary to obtain accurate results [44, 45]. The geNorm program could determine the optimal number of internal reference genes for normalization based on the cut-off of 0.15(Vn/n + 1) [26, 46]. In this study, the V2/3 values for the leaves and roots under salt, alkali, drought, heat, and ABA treatments were below 0.15, indicating that two reference genes would be sufficient under these conditions. According to this rule, it is better to use three genes for cold-treated leaves and roots. However, the 0.15 threshold is not a strict restriction [47]. In addition, considering that the V2/3 values of CL and CR samples were slightly different from 0.15 (0.005 and 0.03), it is not necessary to use three reference genes instead of two as part of the reference gene validation.

Actin genes, including *ACT2/7*, *ACT8*, and *ACT11*, have been widely used as reference genes [38]. In this study, the expression stability of *ACT7* was higher than that of *ACT11* in most of the samples. Moreover, *ACT7* was evaluated as one of the most stably expressed internal reference genes in multiple sample groups, such as SL, SR, AL, and DR samples. In a previous study, *ACT7* was used as the reference gene for normalization of salt response gene expression in *N. sibirica* [2, 48]. This study further demonstrated the reliability of *ACT7* as an internal reference gene for *N. sibirica*. However, the expression stability of *ACT11* was higher than that of *ACT2/7* in soybean. This may be caused by species peculiarity. At the same time, this result also indicates that the stability of individual members of reference gene families can be diverse, and the stability of other members cannot be determined based on one member’s stability. *R3H* was a novel reference gene identified from *N. sibirica* with unknown function and was stably expressed in the SL, DL, ABL, ABR, CL, and HR samples. Some new genes with greater expression stability than traditional housekeeping genes have also been identified in *Lolium multiflorum* [24] and poplar [23]. Therefore, it is not necessary to consider only traditional housekeeping genes when screening stable reference genes. In this study, *EF-1α* was the least stable in AL, DR, CL, HL and HR samples, while the expression of *EF-1α* was one of the most stable reference genes in *Nitraria tangutorum* [13]. This result indicates that the expression level of the same gene may not be constant across multiple species [15]. In addition, *GAPDH* was the best reference gene in the AL, DL, ABL, HL and HR samples of *N. sibirica*, while it exhibited poor stability in SL and AR. The same reference gene in the same species may respond differently to different stresses, as in the case in Kentucky bluegrass [38] and *A. stolonifera* [9]. In summary, it is usually necessary to select reference genes specifically according to species, tissues, and treatments.

Numerous studies have reported significant variations in the expression levels of target genes when unstable reference genes were used as internal controls [41, 49]. To confirm the accuracy of our results, the expression pattern of *NsP5CS* was studied in SL, AL, DR, CR, HL, and ABR samples. The expression of *NsP5CS* in SL, DR, CR and ABR samples was significantly induced, and similar expression patterns of *PuP5CS2*, *AtP5CS1* and *SbP5CS1* under salinity, drought, cold and ABA treatment were reported in *Populus ussuriensis* [50], *Arabidopsis thaliana* [51] and sorghum [52]. The expression of *NsP5CS* in HL samples changed very little, which is consistent with the report that *AtP5CS* is not induced by high temperature [53]. *MsP5CS* was significantly induced by alkaline stress in alfalfa leaves [54], while the expression of *NsP5CS* in our AL samples changed little. This result may be due to the different species and reference genes used in the expression. In addition, as shown in Fig. 5, in each experimental treatment group, if stable reference genes were used to standardize the expression of *NsP5CS*, although there would be some small differences, the expression patterns were very similar. As previous studies have shown, the small differences can usually be corrected with more reference genes [55]. However, if unstable reference genes were used for normalization, the expression pattern of *NsP5CS* would be significantly deviated. The results of the validation test show that the reference genes screened in this study were reliable.

In summary, we conducted a systematic analysis to support the selection of stable reference genes for RT–qPCR analysis in leaves and roots of *N. sibirica* under six different treatments for the first time. *ACT7* combined with *R3H*, *GAPDH*, *TUB* or *His* were the most stable reference genes in SL, SR, AL, DR samples, respectively; *R3H* and *GAPDH* were the most suitable combination for DL and HR; *DIM1* and *His* maintained stable expression in AR sample; *TUB* combined with *R3H* was stable in ABR sample; *TBCB* and *GAPDH* exhibited stable expression in HL group; *TBCB*, *R3H*, and *ERF3A* were stable reference genes in CL group; and for CR sample, the three most stable reference genes were *TBCB*, *ACT11* and *DIM1*. These results will improve the accuracy of target gene expression quantification, facilitate the identification of stress-responsive genes and help to reveal the molecular mechanisms conferring stress tolerance in *N. sibirica*.

## Materials and methods

### Sample preparation and treatments

Seeds of *N. sibirica* were collected from Keluke beach saline–alkaline soil in the Qaidam Basin of Qinghai Province, China (37^◦^ 10’–37^◦^ 20’ N, 96^◦^ 49’–97^◦^ 37’ E), and they were identified by Zhang Huaxin, Institute of Ecological Protection and Restoration, Chinese Academy of Forestry (Voucher: Zhang Faqi, 20,170,823, Kunming Institute of Botany, Chinese Academy of Sciences, China). The seed collection followed relevant institutional, national, and international guidelines and legislation. The seeds were soaked in warm distilled water (40–50 °C) for 24 h to promote germination [56]. After surface sterilization using 10% (m/v) sodium hypochlorite for 15 min, the seeds were sown on Murashige and Skoog (1962; MS) medium containing 3.0% sucrose and 0.68% (m/v) agar. Then, the seeds were cultured at 26 °C, a 14-h photoperiod, and a photon flux density of 45 µmol m^− 2^ s^− 1^. Three-week-old *N. sibirica* seedlings were transplanted into glass test tubes with half-strength Hoagland’s solution that was renewed every week and grown under the same conditions. After 4 weeks, seedlings with good and consistent growth were randomly collected for different experimental treatments. For salt, alkali and drought stress, seedlings were treated with 400 mM NaCl, 120 mM NaHCO_3_ and 20% PEG 6000 (w/v, polyethylene glycol), respectively. For heat and cold stress, the seedlings were subjected to temperatures of 45 °C and 4 °C, respectively, in a lighted incubator. For hormone treatments, the seedlings were treated with 100 µM ABA. The leaves and roots were harvested at 3, 6, 12, 24 and 48 h of treatment. At the same time, leaves and roots of untreated seedlings were also harvested as a blank control. The harvested samples were washed with purified water, immediately frozen in liquid nitrogen and stored at − 80 °C for further analysis. Notably, all the above treatments were performed in three biological replicates, and three seedlings were collected from each replicate.

### Total RNA extraction and cDNA synthesis

RNA extraction was performed using the E.Z.N.A.® Plant Kit (OMEGA Bio-Tek, Doraville, GA, USA) according to the product manual. The integrity of the extracted RNA was assessed by 1.5% agarose gel electrophoresis. Samples with a 28S/18S ratio of approximately 2.0 and no dispersion could be used for subsequent experiments. The quality and purity of the extracted RNA was determined using a NanoDrop 2000 spectrophotometer (Thermo Fisher Scientific, Waltham, MA, USA). The absorbance ratios of the RNA samples at 260/280 and 260/230 nm ranged from 1.8 to 2.0 and 2.0 to 2.4, respectively, indicating suitable quality for subsequent research. According to the HiFiScript gDNA Removal RT MasterMix (CoWin Biosciences, Beijing, China) kit instructions, 1 µg of high-quality RNA extracted from each sample was reverse transcribed into first-strand cDNA. The obtained 20 µL cDNA products were then diluted to a volume of 60 µL for RT–qPCR.

### Candidate reference gene selection and primer design

Candidate genes with stable expression were first selected according to the preliminary *N. sibirica* transcriptome data (NCBI with the ID GSE113246 and PRJNA804704); then, the candidate genes were further screened by combining gene annotation and public reports [13, 45]. Finally, a total of 10 genes were selected as candidate reference genes. These candidate reference genes included 8 traditional reference genes (*ACT7*, *ACT11*, *TUB*, *ERF3A*, *TBCB*, *GAPDH*, *EF-1α* and *His*) and two novel genes, *DIM1* and *R3H.* Primer Premier 5.0 software [57] was used to design the candidate reference gene primers. The parameters were set as follows: melting temperature (Tm), 55–65℃; GC content, 40–60%; primer length, 17–26 bp; and amplification product size, 150–300 bp [21, 58]. All primers (Table 1) were synthesized by RuiBiotech company (Beijing, China). All primers were verified by electrophoresis on a 1.5% agarose gel.

### RT–qPCR conditions and amplification efficiency test

RT–qPCR was performed in 96-well plates by using a LightCycler® 480II Real-Time PCR System (Roche Molecular Systems, Germany). cDNA was amplified by using UItraSYBR Mixture (CoWin Biosciences, Beijing, China). Each reaction mixture contained 10 µl of 2×UItraSYBR Mixture, 0.8 µl of cDNA, 0.4 µl of each primer (10 µM) and 8.4 µl of ddH_2_O. The amplification conditions were as follows: an initial step of 95 °C for 10 min, followed by 40 cycles of 95 °C for 10 s, 55 °C for 30 s and 72 °C for 32 s. The final melting curve was produced by shifting the amplification temperature from 65 to 95 °C. RT–qPCR analysis of each sample was performed in triplicate, and template-free controls were included in parallel. The standard curve was constructed with a tenfold dilution (10, 100, 1000, 10,000) of a cDNA mixture comprising equal volumes of cDNA from all samples, and the amplification efficiency (E) and correlation coefficient (R^2^) values of the primers were calculated using the standard curve. The E value of each primer pair was calculated by the curve slope using E = 10^(−1/slope)^ [59].

### Stability analysis of candidate reference genes

To assess the stability of candidate reference genes, we first analysed the resulting RT–qPCR data using four software programs: geNorm, NormFinder, BestKeeper and comparative ΔCt method. Then, RefFinder (https://www.heartcure.com.au/reffinder/) was used to generate a comprehensive ranking of the candidate reference genes according to data obtained by geNorm, NormFinder, BestKeeper and comparative ΔCt [42]. For geNorm and NormFinder analysis, the raw quantification cycle values (Cq) need to be converted into relative quantities, and for BestKeeper and comparative ΔCt algorithms, raw Cq values could meet the requirements.

### Validation of reference gene stability

To validate the reliability of the selected reference genes, the relative expression level of *NsP5CS* was analysed in SL, AL, DR, CR, HL and ABR samples. The primer pair of *NsP5CS* (Table 1) was designed with Primer Premier 5.0 software. The RT‒qPCR conditions were set up the same as the RT‒qPCR conditions described above. The expression of *NsP5CS* was calculated using (E_target_) ^ΔCq^_target_^(control−sample)^/(E_ref_) ^ΔCq^_ref_^(control−sample)^ [60] with the two worst and two best reference genes (alone and in combination) obtained by the comprehensive assessment used as references.

## Supplementary Information

**Additional file 1: Figure S1, Figure S2, Figure S3 and** **Figure S4**.

**Additional file 2:** **Table S1, Table S2, Table S3, Table S4** **and Table S5**.

## Data Availability

The main data supporting the results of this article are included within the article (and its additional files).

## References

[1] Zhang G (2013). Effects of iso-osmotic salt and water stresses on growth and ionic absorption and distribution in*Nitraria sibirica*seedlings. Agric Res Arid Areas.

[2] Li H, Tang X, Zhu J, Yang X, Zhang H. De novo transcriptome characterization, gene expression profiling and ionic responses of *Nitraria sibirica* Pall. Under salt stress. Forests. 2017;8(6):211.

[3] Song Q, Xia X, Ji C, Chen D, Lu Y (2019). Optimized flash extraction and UPLC-MS analysis on antioxidant compositions of Nitraria sibirica fruit. J Pharm Biomed Anal.

[4] Sa R, chen G (2013). Effect of exogenous spermidine on antioxidant enzyme system in leaves of*Nitraria sibirica*Pall. Seedlings under salt stress. Acta Bot Boreali-Occidentalia Sin.

[5] Chen T, Li H, Wu h, Liu z, Wu X, Yang S (2015). Comparison on osmotica accumulation of different salt-tolerant plants under salt stress. For Res.

[6] Tang X, Zhang H, Shabala S, Li H, Yang X, Zhang H (2021). Tissue tolerance mechanisms conferring salinity tolerance in a halophytic perennial species *Nitraria sibirica* Pall. Tree Physiol.

[7] Derveaux S, Vandesompele J, Hellemans J (2010). How to do successful gene expression analysis using real-time PCR. Methods.

[8] Kanakachari M, Solanke AU, Prabhakaran N, Ahmad I, Dhandapani G, Jayabalan N (2016). Evaluation of suitable reference genes for normalization of qPCR gene expression studies in Brinjal (*Solanum melongena* L.) during Fruit Developmental Stages. Appl Biochem Biotechnol.

[9] Chen Y, Hu B, Tan Z, Liu J, Yang Z, Li Z (2015). Selection of reference genes for quantitative real-time PCR normalization in creeping bentgrass involved in four abiotic stresses. Plant Cell Rep.

[10] Andersen CL, Jensen JL, Orntoft TF (2004). Normalization of real-time quantitative reverse transcription-PCR data: a model-based variance estimation approach to identify genes suited for normalization, applied to bladder and colon cancer data sets. Cancer Res.

[11] Delporte M, Legrand G, Hilbert JL, Gagneul D (2015). Selection and validation of reference genes for quantitative real-time PCR analysis of gene expression in *Cichorium intybus*. Front Plant Sci.

[12] Gutierrez L, Mauriat M, Guénin S, Pelloux J, Lefebvre JF, Louvet R (2008). The lack of a systematic validation of reference genes: a serious pitfall undervalued in reverse transcription-polymerase chain reaction (RT-PCR) analysis in plants. Plant Biotechnol J.

[13] Wang B, Duan H, Chong P, Su S, Shan L, Yi D (2020). Systematic selection and validation of suitable reference genes for quantitative real-time PCR normalization studies of gene expression in *Nitraria tangutorum*. Sci Rep.

[14] Shukla P, Reddy RA, Ponnuvel KM, Rohela GK, Shabnam AA, Ghosh MK (2019). Selection of suitable reference genes for quantitative real-time PCR gene expression analysis in Mulberry (*Morus alba* L.) under different abiotic stresses. Mol Biol Rep.

[15] Sgamma T, Pape J, Massiah A, Jackson S (2016). Selection of reference genes for diurnal and developmental time-course real-time PCR expression analyses in lettuce. Plant Methods.

[16] He Y, Zhong Y, Bao Z, Wang W, Xu X, Gai Y (2021). Evaluation of *Angelica decursiva* reference genes under various stimuli for RT-qPCR data normalization. Sci Rep.

[17] Hu X, Zhang L, Nan S, Miao X, Yang P, Duan G (2018). Selection and validation of reference genes for quantitative real-time PCR in *Artemisia sphaerocephala* based on transcriptome sequence data. Gene.

[18] Yang C-L, Yuan X-Y, Zhang J, Sun W-H, Liu Z-J, Zou S-Q. Comprehensive transcriptome analysis of reference genes for fruit development of *Euscaphis konishii*. PeerJ. 2020;8:e8474. 10.7717/peerj.8474 PMC7020815 32095336

[19] Chang E, Shi S, Liu J, Cheng T, Xue L, Yang X (2012). Selection of reference genes for quantitative gene expression studies in *Platycladus orientalis* (Cupressaceae) using real-time PCR. PLoS ONE.

[20] Galeano E, Vasconcelos TS, Ramiro DA, De Martin Vde F, Carrer H (2014). Identification and validation of quantitative real-time reverse transcription PCR reference genes for gene expression analysis in teak (*Tectona grandis* L.f.). BMC Res Notes.

[21] Wang Y, Zhang Y, Liu Q, Tong H, Zhang T, Gu C (2021). Selection and validation of appropriate reference genes for RT-qPCR analysis of flowering stages and different genotypes of *Iris germanica* L. Sci Rep.

[22] Li L, Li N, Fang H, Qi X, Zhou Y (2020). Selection and validation of reference genes for normalisation of gene expression in *Glehnia littoralis*. Sci Rep.

[23] Chu W, Wang Y, Zhu D, Chen Z, Yan H, Xiang Y (2017). Selection of novel reference genes in Poplar under salt and drought stresses. Sci Silvae Sin.

[24] Liu Q, Qi X, Yan H, Huang L, Nie G, Zhang X. Reference gene selection for quantitative real-time reverse-transcriptase PCR in Annual Ryegrass (*Lolium multiflorum*) subjected to various abiotic stresses. Molecules. 2018;23(1):172. 10.3390/molecules23010172 PMC6017817 29337852

[25] Bakir Y, Eldem V, Zararsiz G, Unver T. Global transcriptome analysis reveals differences in gene expression patterns between nonhyperhydric and hyperhydric peach leaves. Plant Genome. 2016;9(2):0080. 10.3835/plantgenome2015.09.0080 27898837

[26] Vandesompele J, De Preter K, Pattyn F, Poppe B, Van Roy N, De Paepe A (2002). Accurate normalization of real-time quantitative RT-PCR data by geometric averaging of multiple internal control genes. Genome Biol.

[27] Pfaffl MW, Tichopad A, Prgomet C, Neuvians TP (2004). Determination of stable housekeeping genes, differentially regulated target genes and sample integrity: BestKeeper–Excel-based tool using pair-wise correlations. Biotechnol Lett.

[28] Silver N, Best S, Jiang J, Thein SL (2006). Selection of housekeeping genes for gene expression studies in human reticulocytes using real-time PCR. BMC Mol Biol.

[29] Xie F, Xiao P, Chen D, Xu L, Zhang B (2012). miRDeepFinder: a miRNA analysis tool for deep sequencing of plant small RNAs. Plant Mol Biol.

[30] Guan C, Ji J, Guan W, Feng Y, Li X, Jin C (2014). A Lycium chinense-derived P5CS-like gene is regulated by water deficit-induced endogenous abscisic acid and overexpression of this gene enhances tolerance to water deficit stress in *Arabidopsis*. Mol Breeding.

[31] Zheng L, Dang Z, Li H, Zhang H, Wu S, Wang Y (2014). Isolation and characterization of a ∆1-pyrroline-5-carboxylate synthetase (*NtP5CS)* from *Nitraria tangutorum* Bobr. And functional comparison with its *Arabidopsis* homologue. Mol Biol Rep.

[32] Xiao X, Ma J, Wang J, Wu X, Li P, Yao Y (2014). Validation of suitable reference genes for gene expression analysis in the halophyte *Salicornia europaea* by real-time quantitative PCR. Front Plant Sci.

[33] Niu X, Chen M, Huang X, Chen H, Tao A, Xu J (2017). Reference gene selection for qRT-PCR normalization analysis in kenaf (*Hibiscus cannabinus* L.) under abiotic stress and hormonal stimuli. Front Plant Sci.

[34] Bustin S (2002). Quantification of mRNA using real-time reverse transcription PCR (RT-PCR): trends and problems. J Mol Endocrinol.

[35] Taylor S, Wakem M, Dijkman G, Alsarraj M, Nguyen M (2010). A practical approach to RT-qPCR-Publishing data that conform to the MIQE guidelines. Methods.

[36] Abbas A, Yu H, Li X, Cui H, Chen J, Huang P. Selection and validation of reference genes for RT-qPCR analysis in *Aegilops tauschii* (Coss.) Under different abiotic stresses. Int J Mol Sci. 2021;22(20):10017. 10.3390/ijms222011017 PMC8541341 34681677

[37] Wan H, Zhao Z, Qian C, Sui Y, Malik AA, Chen J. Selection of appropriate reference genes for gene expression studies by quantitative real-time polymerase chain reaction in cucumber. Anal Biochem. 2010;399(2):257–61. 10.1016/j.ab.2009.12.008 20005862

[38] Niu K, Shi Y, Ma H (2017). Selection of candidate reference genes for gene expression analysis in Kentucky Bluegrass (*Poa pratensis* L.) under abiotic stress. Front Plant Sci.

[39] Yang Y, Zhao L, Yang G, ZHANG Y, Fu P, Hu J (2022). Selection and validation of reference genes for leaf color phenotype in *‘Maiyuanjinqiu*’, a *Catalpa fargesii* variety, by qRT-PCR. For Res.

[40] Zhao J, Zhou M, Meng Y. Identification and validation of reference genes for RT-qPCR analysis in Switchgrass under heavy metal stresses. Genes (Basel). 2020;11(5):502. 10.3390/genes11050502 PMC7291066 32375288

[41] Feng K, Liu J, Xing G, Sun S, Li S, Duan A (2019). Selection of appropriate reference genes for RT-qPCR analysis under abiotic stress and hormone treatment in celery. PeerJ.

[42] Su W, Yuan Y, Zhang L, Jiang Y, Gan X, Bai Y (2019). Selection of the optimal reference genes for expression analyses in different materials of Eriobotrya japonica. Plant Methods.

[43] Dudziak K, Sozoniuk M, Szczerba H, Kuzdralinski A, Kowalczyk K, Borner A (2020). Identification of stable reference genes for qPCR studies in common wheat (*Triticum aestivum* L.) seedlings under short-term drought stress. Plant Methods.

[44] Niaz Z, Sui Z, Riaz S, Liu Y, Phycology JJJoA (2019). Identification of valid reference genes for the normalization of RT-qPCR gene expression data in *Alexandrium catenella* under different nutritional conditions. J Appl Phycol.

[45] Wang M, Ren T, Marowa P, Du H, Xu Z (2021). Identification and selection of reference genes for gene expression analysis by quantitative real-time PCR in *Suaeda glauca’s* response to salinity. Sci Rep.

[46] Tang F, Chu L, Shu W, He X, Wang L, Lu M (2019). Selection and validation of reference genes for quantitative expression analysis of miRNAs and mRNAs in Poplar. Plant Methods.

[47] Hou S, Zhao T, Yang D, Li Q, Liang L, Wang G, et al. Selection and validation of reference genes for quantitative RT-PCR analysis in Corylus heterophylla Fisch. X Corylus avellana L. Plants (Basel). 2021;10(1):159. 10.3390/plants10010159 PMC7830083 33467497

[48] Li H, Tang X, Yang X, Zhang H (2021). Comprehensive transcriptome and metabolome profiling reveal metabolic mechanisms of *Nitraria sibirica* Pall. To salt stress. Sci Rep.

[49] Yang Z, Zhang R, Zhou Z. Identification and validation of reference genes for gene expression analysis in *Schima superba*. Genes. 2021;12(5):732. 10.3390/genes12050732 PMC8153319 34068362

[50] Wei M, Chen Y, Zhang M, Yang J, Lu H, Zhang X, et al. Selection and validation of reference genes for the qRT-PCR assays of Populus ussuriensis gene expression under abiotic stresses and related ABA treatment. Forests. 2020;11(4):476.

[51] Liang X, Zhang L, Natarajan SK, Becker DF (2013). Proline mechanisms of stress survival. Antioxid Redox Signal.

[52] Su M, Li XF, Ma XY, Peng XJ, Zhao AG, Cheng LQ (2011). Cloning two P5CS genes from bioenergy sorghum and their expression profiles under abiotic stresses and MeJA treatment. Plant Sci.

[53] Yoshiba Y, Kiyosue T, Katagiri T, Ueda H, Mizoguchi T, Yamaguchi-Shinozaki K (1995). Correlation between the induction of a gene for delta 1-pyrroline-5-carboxylate synthetase and the accumulation of proline in Arabidopsis thaliana under osmotic stress. Plant J.

[54] Song T, Sun N, Dong L, Cai H (2021). Enhanced alkali tolerance of rhizobia-inoculated alfalfa correlates with altered proteins and metabolic processes as well as decreased oxidative damage. Plant Physiol Biochem.

[55] Bustin SA, Benes V, Garson JA, Hellemans J, Huggett J, Kubista M (2009). The MIQE guidelines: minimum information for publication of quantitative real-time PCR experiments. Clin Chem.

[56] Hao M, Wei C, Ren A, Li P (2015). Seedling raising technique of *Nitraria* plants in Yellow River Delta. Sci Technol.

[57] Liu Y, Zhang Y, Liu F, Liu T, Chen J, Fu G (2022). Establishment of reference (housekeeping) genes via quantitative real-time PCR for investigation of the genomic basis of abiotic stress resistance in *Psammochloa villosa* (Poaceae). J Plant Physiol.

[58] Zhang Y, Zhu L, Xue J, Yang J, Hu H, Cui J (2021). Selection and verification of appropriate reference genes for expression normalization in *Cryptomeria fortunei* under abiotic stress and hormone treatments. Genes (Basel).

[59] Meuer S, Wittwer C, Nakagawara K (2001). Rapid cycle real-time PCR: methods and applications.

[60] Pfaffl MW (2001). A new mathematical model for relative quantification in real-time RT-PCR. Nucleic Acids Res.

