# Supplementary material for: Selection and validation of appropriate reference genes for RT–qPCR analysis of Nitraria sibirica under various abiotic stresses
Source: BMC Plant Biol. 2022 Dec 17;22:592. doi: 10.1186/s12870-022-03988-w (PMC9758788; doi:10.1186/s12870-022-03988-w)
Supplement: Supplementary file 1 — Additional file 1: Figure S1, Figure S2, Figure S3 and Figure S4. [file 12870_2022_3988_MOESM1_ESM.docx]

**Selection and validation of appropriate reference genes** **for RT–qPCR analysis of** ***Nitraria sibirica* under various abiotic stresses**

AiShuang Hu^1,2,3^, Xiuyan Yang^1,2^, Jianfeng Zhu^1,2^, Jiaxin Liu^1,2^, Jiping Wang^1,2^, Haiwen Wu^1,2^, Huilong Zhang^1,2*^ & Huaxin Zhang^1,2*^

Supplementary


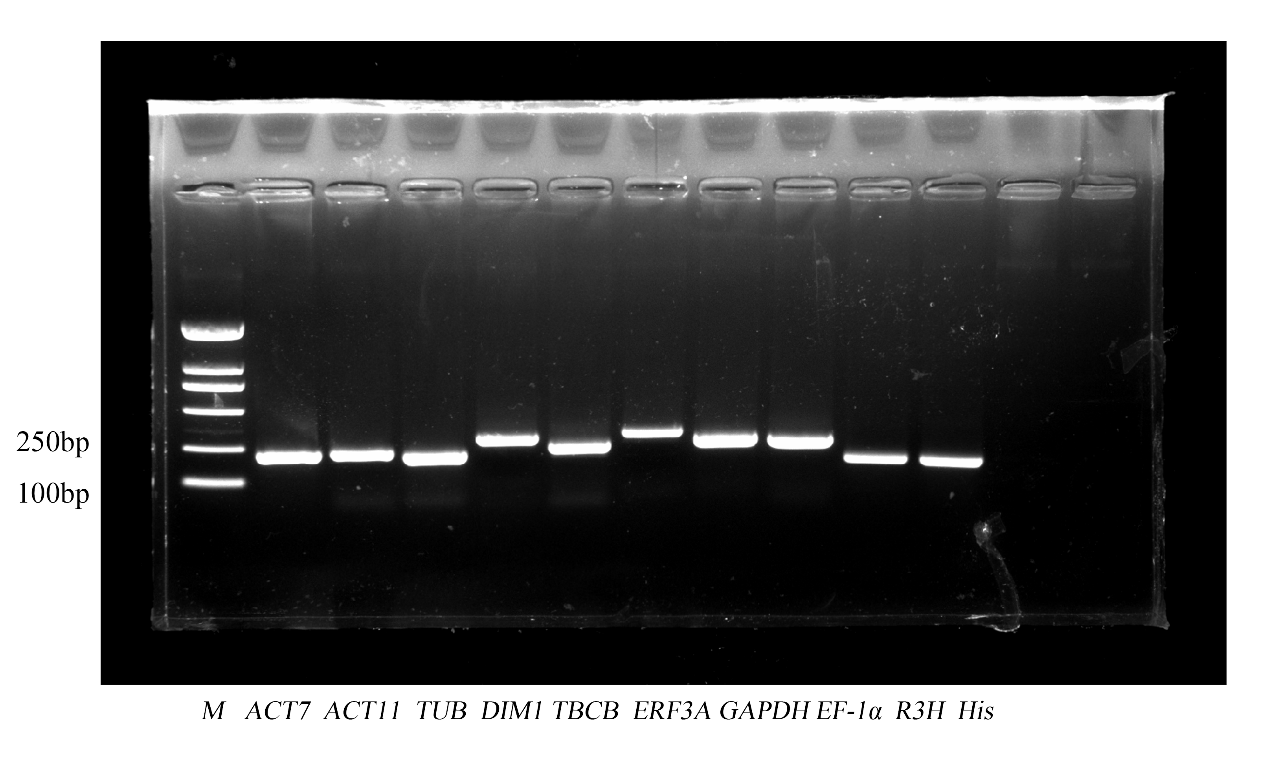
Figure S1. 1.5% agarose gel showing that all primer pairs exhibited specificity and that the sizes of the amplification products were consistent with the expected sizes. M: DL 2000 marker. The names of candidate reference genes were listed at the beginning of each lane


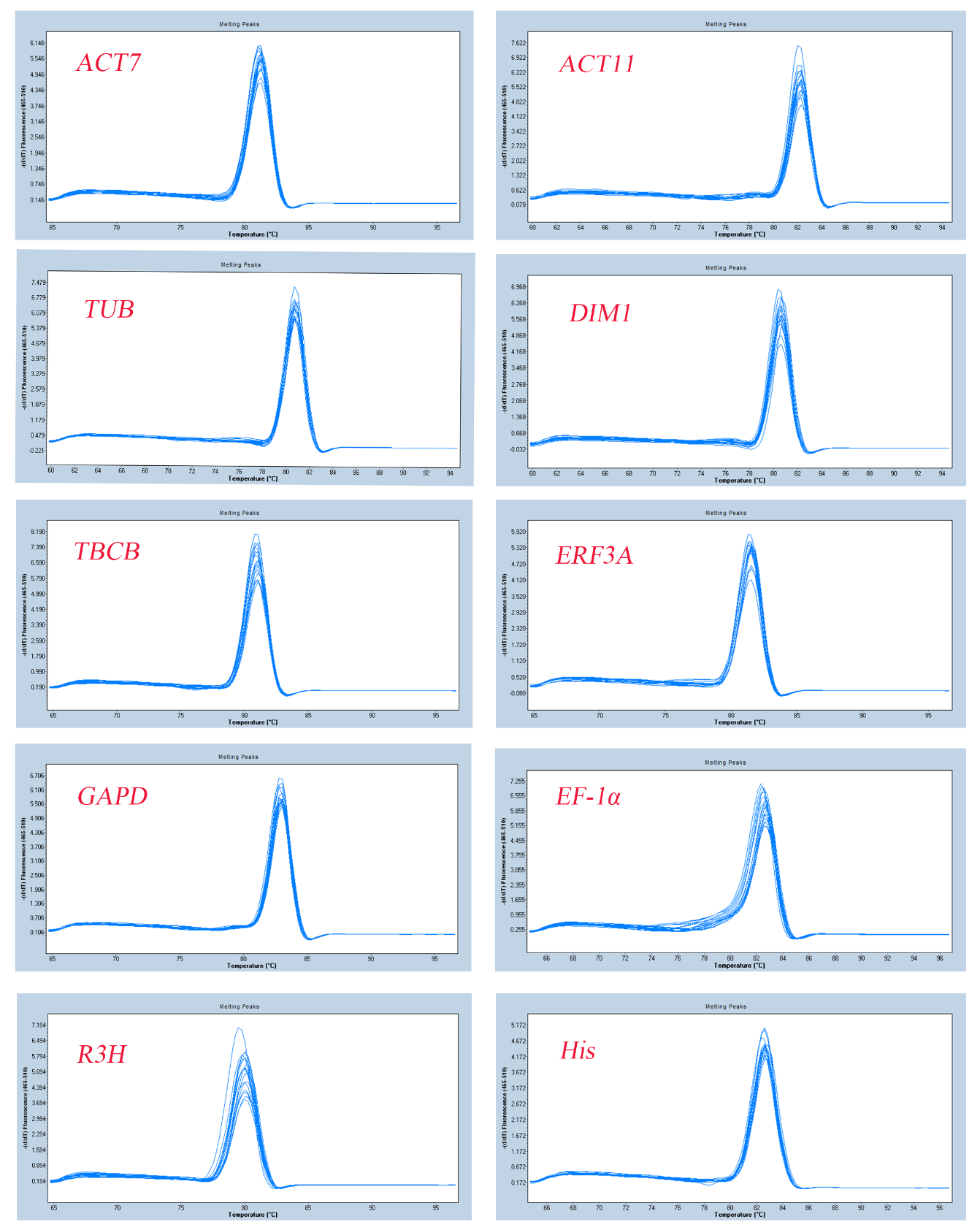


Figure S2. Melting curves of 10 candidate reference genes tested in this study.





Figure S3. The raw Cq values for 10 candidate reference genes in leaves and roots of *N. sibirica*. L-gene and R-gene: gene in leaves and roots. The box indicates the 25th to 75th percentiles, the line across the box represents the median, the inner square represents the mean, and the whiskers represent the maximum and minimum values. the same as below.





Figure S4. The raw Cq values for 10 candidate reference genes of *N. sibirica* under 6 treantments.

1-6: *ACT7* under salt, alkali, drought, cold, heat and ABA treatments; 7-12: *ACT11* under salt, alkali, drought, cold, heat and ABA treatments; 13-18: *TUB* under salt, alkali, drought, cold, heat and ABA treatments; 19-24: *DIM1* under salt, alkali, drought, cold, heat and ABA treatments; 25-30: *TBCB* under salt, alkali, drought, cold, heat and ABA treatments; 31-36: *ERF3A* under salt, alkali, drought, cold, heat and ABA treatments; 37-42: *GAPDH* under salt, alkali, drought, cold, heat and ABA treatments; 43-48: *EF-1α* under salt, alkali, drought, cold, heat and ABA treatments; 49-54: *R3H* under salt, alkali, drought, cold, heat and ABA treatments; 55-60: *His* under salt, alkali, drought, cold, heat and ABA treatments.
